# Genetic relatedness of the *Enterococcus faecalis* isolates in stool and urine samples of patients with community-acquired urinary tract infection

**DOI:** 10.1186/s13099-020-00380-7

**Published:** 2020-09-09

**Authors:** Zohreh Ghalavand, Masoud Alebouyeh, Kiandokht Ghanati, Leila Azimi, Marjan Rashidan

**Affiliations:** 1 School of Medicine, Shahroud University of Medical Sciences, Shahroud, Iran; 2 Pediatric Infections Research Center, Research Institute for Children’s Health, Shahid Beheshti University of Medical Sciences, Tehran, Iran; 3 National Nutrition and Food Technology Research Institute, Shahid Beheshti University of Medical Sciences and Health Services, Tehran, Iran; 4 Department of Microbiology, School of Medicine, Shahid Beheshti University of Medical Sciences, Tehran, Iran

**Keywords:** *Enterococcus faecalis*, Virulence, Urinary tract infection, Fecal microbiota, Antimicrobial resistance

## Abstract

**Background:**

Community-acquired urinary tract infection (CA-UTI) could be caused by endogenous or exogenous routes. To show this relationship, we investigated molecular fingerprints and genotypes of paired *Enterococcus faecalis* isolated from the urine of symptomatic patients and their fecal samples.

**Results:**

Out of the studied patients, 63 pairs of *E. faecalis* isolates were obtained simultaneously from their urine and feces samples. All the strains were sensitive to vancomycin, linezolid, nitrofurantoin, and daptomycin (MIC value: ≤ 4 µg/ml), while resistance to tetracycline (urine: 88.9%; stool: 76.2%) and minocycline (urine: 87.3%, stool: 71.4%) was detected in most of them. The most common detected virulence genes were included *efbA*, *ace*, and *gelE*. RAPD-PCR and PFGE analyses showed the same patterns of molecular fingerprints between paired of the isolates in 26.9% and 15.8% of the patients, respectively.

**Conclusions:**

Similarity of *E. faecalis* strains between the urine and feces samples confirmed the occurrence of endogenous infection via contamination with colonized bacteria in the intestinal tract. Carriage of a complete virulence genotype in the responsible strains was statistically in correlation with endogenous UTI, which shows their possible involvement in pathogenicity of uropathogenic *E. faecalis* strains.

## Background

Urinary tract infections (UTIs) are the most common bacterial infections both in the community and hospital settings at all age groups. Although uropathogenic *Escherichia coli* is the most common cause of community-acquired urinary tract infections in humans [1], *Enterococcus* species, especially *Enterococcus faecalis* (*E. faecalis*), are considered as the second most important cause of UTI among uropathogenic bacteria [2, 3]. *E. faecalis* can also cause surgical wound infection, bacteremia, endocarditis, neonatal sepsis, and meningitis [4]. *E. faecalis* is predominantly inhabitant of the human gastrointestinal tract, where they form part of the normal intestinal flora in approximate amounts of 10^8^ colonies per gram of feces [5]. This rate of colonization could predispose our urinary tract to recurrent infections via the perineal urethral route. This type of infection, which is known as community-acquired urinary tract infection (CA-UTI), is generally attributed to women. This infection may be host-specific, due to the existence of receptors for bacterial adhesins, or mediated by potent virulence factors that are necessary for their pathogenesis in the urinary tract [6].

Management of CA-UTI involves the administration of antibiotics based on susceptibility patterns of responsible bacteria in each region. Prompt elimination of the infection is needed to avoid severe complications in infected patients [6].

Some virulence factors have been proposed for *E. faecalis* to describe its involvement in UTI; however, the pathogenesis of this bacterium and its link with symptoms and complications of the infection is unclear yet. These virulence determinants, such as aggregation substance (*asa1*), gelatinase (*gelE*), cytolysin (*cylA*), enterococcal surface protein (*esp*), collagen-binding-protein (*ace*) and PavA-like fibronectin-binding protein (*efbA*), could facilitate initial colonization, biofilm formation, destruction of the host tissue, and evasion from host immune response. While in the hospitals, factors, such as the use of indwelling medical devices, can facilitate colonization of the urinary tract [6]; however, few data exist about mechanisms that are employed by this bacterium for its colonization in non-hospital settings. Diversity in colonization rate among different strains of this bacterium in different tissues and their pathogenicity could explain the degree of complications that are occurring in the infected patients. While Enterococcal surface protein (Esp), adhesion to collagen of *E. faecalis* (ACE), aggregation substance (AS), PavA-like fibronectin-binding protein (EfbA), cytolysin (CYL), and gelatinase (GelE) are proposed as main virulence factors of *E. faecalis*, no virulence genotype has been suggested for discrimination of the pathogenic from non-pathogenic strains [7–9]. Comparison of phenetic, genomic, and virulence characteristics of the strains causing UTI with those unable to cause this infection could provide more data about this link. This study was aimed to investigate the diversity of virulence determinants, antibiotic resistance profiles, and the genomic relationship of *E. faecalis* strains in urine samples of symptomatic patients with community-acquired UTI compared with those isolated from their stool samples.

## Results

### Patients and clinical isolates of E. faecalis

A total of 126 *E. faecalis* isolates were obtained from 63 patients with CA-UTI. Of these, 63 were derived from urine and 63 were from fecal specimens, simultaneously.). The isolates showed positive results for esculin hydrolysis, 6.5% NaCl, non-fermentation of arabinose, and catalase tests and their identity were confirmed by species specific PCR assay. The mean age for the studied patients was 43 years, which ranged between 6 and 87 years old. The percentage of *E. faecalis* UTI in 63 patients varies across age group, 52.4% female and 47.6% men. Most of the *E. faecalis* isolates were obtained from patients aged between 30 and 60 years old (39/63, 62%).

### Antimicrobial resistance patterns among E. faecalis strains

Susceptibility of *E. faecalis* strains to various antibiotics is shown in Table 1. In general, highest resistance rates were orderly observed against tetracycline (urine: 88.9%, 56/63; stool: 76.2%, 48/63) and minocycline (urine: 87.3%, 55/63; stool: 71.4%, 45/63). No resistance was detected to vancomycin, ampicillin, penicillin, nitrofurantoin, and linezolid in the urine and feces isolates. All the studied strains were susceptible to daptomycin (MIC value: ≤ 4 µg/ml). Except for minocycline, no significant difference was detected between the resistance rates in the strains collected from the urine and stool samples. Comparison of pairs of the isolates from urine and feces specimens showed same resistance patterns among 39 patients (61.9%); however, 17 (26.9%) and 8 (12.6%) pairs of them showed the difference in resistance phenotype to one and greater classes of antimicrobials, respectively (Table 2). A multi-drug resistance (MDR) phenotype was detected in two pairs of isolates. This phenotype was more common in urine samples of the patients with CA-UTIs originating from unrelated strains to the intestinal tract (11.1%, 5/63). All the MDR strains showed tetracyclines/gentamicin (120 µg)/ciprofloxacin/levofloxacin/gatifloxacin resistance patterns.

**Table 1:** Resistance rates to antimicrobials in *E. faecalis* isolates from urine and fecal specimens in patients with community acquired-UTIs

Antibiotics	Urine samples N = 63 (%)			Fecal samples N = 63 (%)		
	R	I	S	R	I	S
Ampicillin (10 µg)	0 (0%)	–	63 (100%)	0 (0%)	–	63 (100%)
Penicillin G (10 units)	0 (0%)	–	63 (100%)	0 (0%)	–	63 (100%)
Vancomycin (30 µg)	0 (0%)	–	63 (100%)	0 (0%)	–	63 (100%)
Linezolide (30 µg)	0 (0%)	–	63 (100%)	0 (0%)	–	63 (100%)
Nitrofurantoin (300 µg)	0 (0%)	–	63 (100%)	0 (0%)	–	63 (100%)
Gatifloxacin (5 µg)	8 (12.7%)	–	55 (87.3%)	4 (6.3%)	–	59 (93.7%)
Levofloxacin (5 µg)	9 (14.3%)	–	54 (85.7%)	4 (6.3%)	–	59 (93.7%)
Ciprofloxacin (5 µg)	13 (20.6%)	–	50 (79.4%)	8 (12.7%)	–	55 (87.3%)
Gentamycin (120 µg)	18 (28.6%)	–	45 (71.4%)	10 (15.9%)	–	53 (84.1%)
Minocycline (30 µg)	55 (87.3%)	–	8 (12.7%)	45 (71.4%)	–	18 (28.6%)
Tetracycline (30 µg)	56 (88.9%)	–	7 (11.1%)	48 (76.2%)	–	15 (23.8%)
Daptomycin	0 (0%)	–	63 (100%)	0 (0%)	–	63 (100%)

Resistance phenotypes were determined for all antibiotics, except daptomycin, by disk diffusion (Kirby–Bauer) method according to CLSI 2014 guidelines (MastGroupLtd, United Kingdom). Resistance to daptomycinwas tested using E-tests trip (Liofilchem^®^, Italy)

*R* resistant, *I* intermediary, *S* susceptible

**Table 2:** Antibiotic resistance patterns in *E. faecalis* isolates form urine and fecal specimens inpatients with community acquired-UTIs

Antibiotic resistance patterns^a^	Urine samples N = 63 (%)	Fecal samples N = 63 (%)	Same resistance patterns^b^
TET, MIN, GM120, CP, LEV, GAT	5 (7.9%)	2 (3.1%)	2
TET, MN, GM120, CP	2 (3.1%)	0 (0%)	0
TET, MN, GM120	10 (15.8%)	8 (12.6%)	7
TET, MN, CP, LEV, GAT	3 (4.7%)	0 (0%)	0
TET, MN, CP, GAT	0 (0%)	1 (1.5%)	0
TET, MN, CP, LEV	0 (0%)	1 (1.5%)	0
TET, CP, LEV, GAT	0 (0%)	1 (1.5%)	0
TET, MN, CP	2 (3.1%)	2 (3.1%)	1
TET, MN	33 (52.3%)	31 (49.2%)	25
CP, LEV	1 (1.5%)	0 (0%)	0
TET	1 (1.5%)	2 (3.1%)	1
GM120	1 (1.5%)	0 (0%)	0
No resistance	4 (6.3%)	15 (23.8%)	3

^a^TET, tetracycline; MIN, minocycline; GM120, gentamicin 120 µg; CP, ciprofloxacin; LEV, levofloxacin; GAT, gatifloxacin. Resistance phenotypes were determined for all antibiotics, except daptomycin, by disk diffusion (Kirby–Bauer) method according to CLSI 2014 guidelines (MastGroupLtd, United Kingdom). Antibiotic concentration for each disk was as follows: Penicillin G (10 units), ampicillin (10 µg), vancomycin (30 µg), tetracycline (30 µg), minocycline (30 µg), ciprofloxacin (5 µg), levofloxacin (5 µg), gatifloxacin (5 µg), nitrofurantoin (300 µg), high level gentamicin-resistant enterococci (HLGRE, 120 µg) and linezolid (30 µg)

^b^Patients with similar resistance patterns in both fecal and urine samples

### Prevalence of virulence determinants in urine and fecal specimens

Analysis of putative virulence determinants among pairs of *E. faecalis* strains showed no significant difference between the fecal and urine isolates in the studied patients The most common detected virulence genes were included *efbA* (100% urine; 96% stool), *ace* (92.1% urine; 96.8% stool), and *gelE* (90.5% urine; 95.2% stool), followed by *asa* (79.4% urine; 65.1% stool), *esp* (77.8% urine; 74.6% stool) and *cyl* (54% urine; 46% stool). The same genotypes were detected among 53 (84.1%) pairs of the isolates, which *esp/efbA/asa1/ace/cyl/gelE* was the commonest genotype among them (Table 3).

**Table 3:** Prevalence of combined virulence determinants among *E. faecalis* isolates in urine and feces specimens of patients with community acquired-UTIs

Genotype patterns	Urine samples N = 63 (%)	Fecal samples N = 63 (%)
*esp*^+^*, efbA*^+^*, asa*^+^*, ace*^+^*, cyl*^+^*, gelE*^+^	25 (39.6%)	23 (36.5%)
*esp*^+^*, efbA*^+^*, asa*^+^*, ace*^+^*, cyl*^+^	6 (9.5%)	1 (1.5%)
*esp*^+^*, efbA*^+^*, asa*^+^*, ace*^+^*, gelE*^+^	9 (14.2%)	7 (11.1%)
*esp*^+^*, efbA*^+^*, ace*^+^*, cyl*^+^*, gelE*^+^	1 (1.5%)	3 (4.7%)
*efbA*^+^*, asa*^+^*, ace*^+^*, cyl*^+^*, gelE*^+^	2 (3.1%)	0 (0%)
*esp*^+^*, efbA*^+^*, asa*^+^*, cyl*^+^*, gelE*^+^	0 (0%)	1 (1.5%)
*efbA*^+^*, asa*^+^*, ace*^+^*, gelE*^+^	8 (12.6%)	9 (14.2%)
*esp*^+^*, efbA*^+^*, ace*^+^*, gelE*^+^	7 (11.1%)	10 (15.8%)
*efbA*^+^*, ace*^+^*, cyl*^+^*, gelE*^+^	1 (1.5%)	1 (1.5%)
*efbA*^+^*, ace*^+^*, gelE*^+^	4 (6.3%)	4 (6.3%)
*esp*^+^*, efbA*^+^*, gelE*^+^	1 (1.5%)	1 (1.5%)
*esp*^+^*, efbA*^+^*, ace*^+^	0 (0%)	1 (1.5%)
*ace*^+^*, gelE*^+^	0 (0%)	1 (1.5%)

+ , gene present

*esp,* Enterococcal surface protein; *asa1*, Aggregation substance; *ace*, Adhesion of collagen of enterococci; *cyl, *Cytolysin; *gelE*, Gelatinase; *efbA*, Pav A-like fibronectin-binding protein

### Association of antibiotic resistance patterns and virulence determinants among E. feacalis isolates

A comparison of the results of the urine and feces isolates of each patient indicated that 17 out of 21 pairs of the strains with the same resistance phenotypes also presented same virulence genotypes. Consistency of the resistance phenotypes and virulence genotypes was associated with the strains that depicted T/MN/CIP/GEN(120 µg)/LEV/GAT (100%, 2/2), T/MN/GEN(120 µg)(42.8%, 3/7), T/MN/CIP (100%, 1/1), and TET/MN (60%, 15/25) resistance patterns.

### DNA fingerprinting analysis techniques

#### RAPD-PCR

RAPD-PCR was performed for the 126 isolates (63 urine and 63 feces). A comparison of RAPD-PCR electrophoretic patterns indicated 17 pairs of the strains (26.9%) with similar genotypic patterns. Out of them, 11 pairs of the strains (64.7%) showed complete genotype (*esp*^+^*/efbA*^+^*/asa1*^+^*/ace*^+^*/cyl*^+^*/gelE*^+^) and 6 pairs (35.2%) showed partial genotypes (Table 4). All UTIs that were caused by *E. faecalis* strains with identical RAPD, drug resistance, and virulence genotype patterns compared with those isolated from the fecal samples in the same patients were defined as endogenous infection. Conversely, the exogenous infection was detected in 46 (73%) samples. Genotypic patterns of these strains are shown in Table 4. There was a significant correlation between the complete genotype and the determined endogenous infection based on the RAPD patterns (*p-*value = 0.02).

**Table 4:** Association of genotypic patterns with endogenous and exogenous infections in *E. faecalis* strains using RAPD-PCR assay

Genotypic patterns	Endogenous UTIs n = 17	Exogenous UTIs n = 46	Total	*p* value
*esp*^+^*, efbA*^+^*, asa*^+^*, ace*^+^*, cyl*^+^*, gelE*^+^	11 (64.7%)	14 (30.4%)	25	0.02
*esp*^+^*, efbA*^+^*, asa*^+^*, ace*^+^*, cyl*^+^	0 (0%)	6 (13%)	6	*0.17*
*esp*^+^*, efbA*^+^*, asa*^+^*, ace*^+^*, gelE*^+^	1 (5.8%)	9 (19.5%)	10	*0.26*
*esp*^+^*, efbA*^+^*, ace*^+^*, cyl*^+^*, gelE*^+^	0 (0%)	1 (2.1%)	1	*1*
*efbA*^+^*, asa*^+^*, ace*^+^*, cyl*^+^*, gelE*^+^	0 (0%)	2 (4.3%)	2	*1*
*efbA*^+^*, asa*^+^*, ace*^+^*, gelE*^+^	2 (11.7%)	6 (13%)	8	*1*
*esp*^+^*, efbA*^+^*, ace*^+^*, gelE*^+^	3 (17.6%)	4 (8.6%)	7	*0.39*
*efbA*^+^*, ace*^+^*, gelE*^+^	0 (0%)	4 (8.6%)	4	*0.56*
*esp*^+^*, efbA*^+^*, gelE*^+^	0 (0%)	1 (2.1%)	1	*1*

+ , gene present. Italic face indicates values that are significant (*p* < 0.05)

*esp,* Enterococcal surface protein; *asa1*, Aggregation substance; *ace*, Adhesion of collagen of enterococci; *cyl, *Cytolysin; *gelE*, Gelatinase; *efbA*, Pav A-like fibronectin-binding protein

#### PFGE

**Fig. 1:**
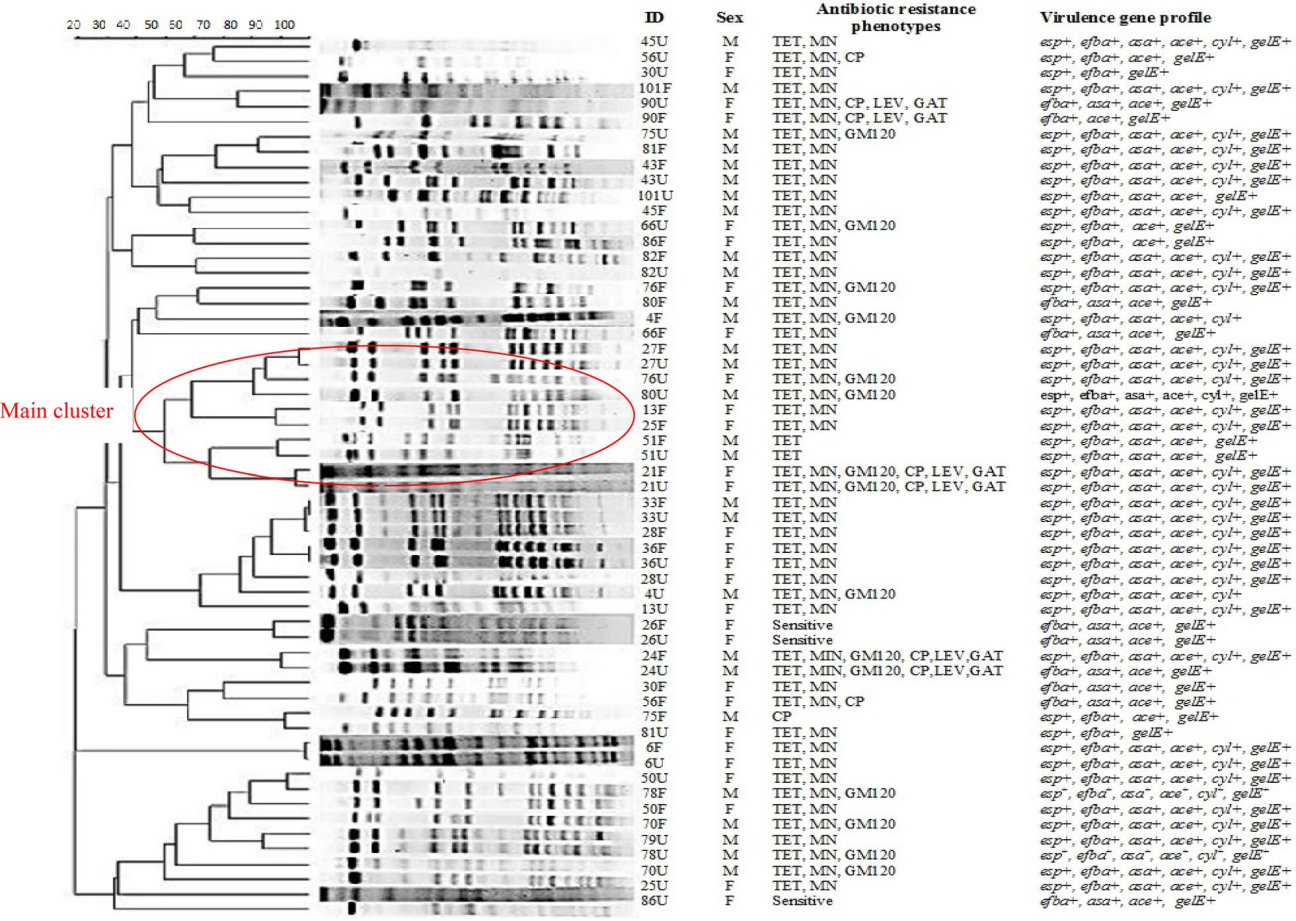
Pulse field gel electrophoresis of *E. faecalis* strains in patients with CA-UTI. First column: Patients’ code plussource of each isolate, F: Feces, U: urine; Second column: F, Female; M, Male; Third column, antibiotic resistance phenotype: TET, tetracycline; MIN, minocycline; GM120, gentamicin 120 µg; CP, ciprofloxacin; LEV, levofloxacin; GAT, gatifloxacin; Fourth column, virulence factors: *esp,* Enterococcal surface protein; *asa1*, Aggregation substance; *ace*, Adhesion of collagen of enterococci; *cyl,* Cytolysin; *gelE*, Gelatinase; *efbA*, Pav A-like fibronectin-binding protein. The PFGE patterns were determined using the Dice coefficient in GelCompar II version 2.0 (Applied Maths, Belgium). Isolates that differed by ≤ 3 bands were assigned to same pulse type (PT), while isolates that differed by ≥ 4 bands assigned to different types

According to the results of RAPD-PCR, pairs of the isolates (34 pairs with similar RAPD types and 24 pairs with different RAPD types) were selected for PFGE analysis. Considering a cut off value of 87%, eleven strains (18.9%) showed common pulsotypes (CT) and 32 strains (55%) were singletons (ST). Amongst the CT, 10 pairs of the strains from the urine and fecal samples showed similar pulsotypes (Fig. 1). The characterized pulsotypes in patients with endogenous infections and their link with antibiotic resistance patterns and virulence determinants are shown in Fig. 1 and Table 5. Comparison of *E. faecalis* isolates in urine and stool samples of patients with exogenous UTI based on antibiotic resistance patterns and virulence determinants are shown in Table 6.

**Table 5:** Comparison of *E. faecalis* isolates in urine and stool samples of patients with endogenous UTI based on antibiotic resistance patterns, virulence determinants, and Pulse types

Antibiotic resistance patterns		Virulence determinant patterns		Pulsotype	No. of patients
Urine	Feces	Urine	Feces		
TET/MN	TET/MN	*esp*^+^*, efbA*^+^*, asa*^+^*, ace*^+^*, cyl*^+^*, gelE*^+^	*esp*^+^*, efbA*^+^*, asa*^+^*, ace*^+^*, cyl*^+^*, gelE*^+^	Identical	6
TET/MN/GM(120)/CP/GAT/LEV	TET/MN/GM(120)CP/GAT/LEV	*esp*^+^*, efbA*^+^*, asa*^+^*, ace*^+^*, cyl*^+^*, gelE*^+^	*esp*^+^*, efbA*^+^*, asa*^+^*, ace*^+^*, cyl*^+^*, gelE*^+^	Identical	1
TET/MN/GM(120)/CP/GAT/LEV	TET/MN/GM(120)/CP/GAT/LEV	*efbA*^+^* ,asa*^+^*, ace*^+^*, gelE*^+^	*efbA*^+^*, asa*^+^*,ace*^+^*, gelE*^+^	Identical	1
Sensitive to all antibiotics	Sensitive to all antibiotics	*efbA*^+^*, asa*^+^*, ace*^+^*, gelE*^+^	*efbA*^+^*, asa*^+^*, ace*^+^*, gelE*^+^	Identical	1
TET	TET	*esp*^+^*, efbA*^+^*, ace*^+^*, gelE*^+^	*esp*^+^*, efbA*^+^*, asa*^+^*, ace*^+^*, gelE*^+^	Identical	1

+ , gene present

No. of patients: Number of patients; TET, tetracycline; MIN, minocycline; GM120, gentamicin 120 µg; CP, ciprofloxacin; LEV, levofloxacin; GAT, gatifloxacin; *esp,* Enterococcal surface protein; *asa1*, Aggregation substance; *ace*, Adhesion of collagen of enterococci; *cyl, *Cytolysin; *gelE*, Gelatinase; *efbA*, Pav A-like fibronectin-binding protein

**Table 6:** Comparison of *E. faecalis* isolates in urine and stool samples of patients with exogenous UTI based on antibiotic resistance patterns and virulence determinants

Antibiotic resistance patterns		Virulence determinant patterns		No. of patients
Urine	Feces	Urine	Feces	
TET/MN	TET/MN	*esp*^+^*, efbA*^+^*, asa*^+^*, ace*^+^*, cyl*^+^*, gelE*^+^	*esp*^+^*, efbA*^+^*, asa*^+^*, ace*^+^*, cyl*^+^*, gelE*^+^	7
TET/MN/GM(120)	TET/MN/GM(120)	*esp*^+^*, efbA*^+^*, asa*^+^*, ace*^+^*, cyl*^+^*, gelE*^+^	*esp*^+^*, efbA*^+^*, asa*^+^*, ace*^+^*, cyl*^+^*, gelE*^+^	2
TET/MN/GM(120)	TET/MN/CP	*esp*^+^*, efbA*^+^*, asa*^+^*, ace*^+^*, cyl*^+^*, gelE*^+^	*esp*^+^*, efbA*^+^*, asa*^+^*, ace*^+^*, cyl*^+^*, gelE*^+^	1
TET/MN	TET/MN/CP/GAT/LEV	*esp*^+^*, efbA*^+^*, asa*^+^*, ace*^+^*, cyl*^+^*, gelE*^+^	*esp*^+^*, efbA*^+^*, asa*^+^*, ace*^+^*, cyl*^+^*, gelE*^+^	1
TET/MN/GM(120)/CP	CP	*esp*^+^*, efbA*^+^*, asa*^+^*, ace*^+^*, cyl*^+^*, gelE*^+^	*esp*^+^*, efbA*^+^*, asa*^+^*, ace*^+^*, cyl*^+^*, gelE*^+^	1
TET/MN	TET/MN	*esp*^+^*, efbA*^+^*, ace*^+^*, gelE*^+^	*esp*^+^*, efbA*^+^*, ace*^+^*, gelE*^+^	1
TET/MN/GM(120)/CP/GAT/LEV	TET/MN	*esp*^+^*, efbA*^+^*, ace*^+^*, gelE*^+^	*esp*^+^*, efbA*^+^*, ace*^+^*, gelE*^+^	1
TET/MN	Sensitive to all antibiotics	*esp*^+^*, efbA*^+^*, ace*^+^*, gelE*^+^	*esp*^+^*, efbA*^+^*, ace*^+^*, gelE*^+^	1
TET/MN/CP	TET/MN/CP	*esp*^+^*, efbA*^+^*, ace*^+^*, gelE*^+^	*esp*^+^*, efbA*^+^*, ace*^+^*, gelE*^+^	1
TET/MN/CP/GAT/LEV	TET/MN	*efbA*^+^*,asa*^+^*,ace*^+^*, gelE*^+^	*efbA*^+^*, asa*^+^*,ace*^+^*, gelE*^+^	1
TET/MN	TET/MN	*efbA*^+^*,asa*^+^*,ace*^+^*, gelE*^+^	*efbA*^+^*,asa*^+^*,ace*^+^*, gelE*^+^	1
Sensitive to all antibiotics	Sensitive to all antibiotics	*efbA*^+^*,asa*^+^*,ace*^+^*, gelE*^+^	*efbA*^+^*,asa*^+^*,ace*^+^*, gelE*^+^	1
TET/MN/GM(120)/CP	TET/MN/GM(120)	*esp*^+^*, efbA*^+^*, asa*^+^*, ace*^+^*, cyl*^+^	*esp*^+^*, efbA*^+^*, asa*^+^*, ace*^+^*, gelE*^+^	1
TET/MN	TET/MN	*esp*^+^*, efbA*^+^*, asa*^+^*, ace*^+^*, gelE*^+^	*esp*^+^*, efbA*^+^*, asa*^+^*, ace*^+^*, gelE*^+^	1
TET/MN/CP	TET/MN	*esp*^+^*, efbA*^+^*, asa*^+^*, ace*^+^*, gelE*^+^	*esp*^+^*, efbA*^+^*, asa*^+^*, ace*^+^*, gelE*^+^	1
Sensitive to all antibiotics	TET	*esp*^+^*, efbA*^+^*, asa*^+^*, ace*^+^*, gelE*^+^	*esp*^+^*, efbA*^+^*, asa*^+^*, ace*^+^*, gelE*^+^	1
TET/MN/GM(120)	TET/MN/GM(120)	*esp*^+^*, efbA*^+^*, asa*^+^*, ace*^+^*, cyl*^+^	*esp*^+^*, efbA*^+^*, asa*^+^*, ace*^+^*, cyl*^+^	1
TET/MN/CP/GAT/LEV	Sensitive to all antibiotics	*esp*^+^*, efbA*^+^*, asa*^+^*, ace*^+^*, gelE*^+^	*esp*^+^*, efbA*^+^*, ace*^+^*, gelE*^+^	1
TET/MN/GM(120)	TET/MN/GM(120)	*esp*^+^*, efbA*^+^*, asa*^+^*, ace*^+^*, gelE*^+^	*esp*^+^*, efbA*^+^*, asa*^+^*, ace*^+^*, gelE*^+^	1
TET/MN/GM(120)	Sensitive to all antibiotics	*efbA*^+^*, asa*^+^*, ace*^+^*, cyl*^+^*, gelE*^+^	*efbA*^+^*, ace*^+^*, gelE*^+^	1
TET/MN	TET/MN	*efbA*^+^*, asa*^+^*, ace*^+^*, cyl*^+^*, gelE*^+^	*efbA*^+^*, ace*^+^*, gelE*^+^	1
TET/MN	TET/MN	*esp*^+^*, efbA*^+^*, ace*^+^*, gelE*^+^	*efbA*^+^*, asa*^+^*, ace*^+^*, gelE*^+^	1
TET/MN/GM(120)	TET/MN	*esp*^+^*, efbA*^+^*, ace*^+^*, gelE*^+^	*efbA*^+^*, asa*^+^*, ace*^+^*, gelE*^+^	1
TET/MN	Sensitive to all antibiotics	*esp*^+^*, efbA*^+^*, ace*^+^*, gelE*^+^	*efbA*^+^*, asa*^+^*, ace*^+^*, gelE*^+^	1
TET/MN	TET/MN	*esp*^+^*, efbA*^+^*, asa*^+^*, ace*^+^*, gelE*^+^	*esp*^+^*, efbA*^+^*, asa*^+^*, ace*^+^*, cyl*^+^*, gelE*^+^	1
TET/MN/GM(120)	TET/MN/GM(120)	*esp*^+^*, efbA*^+^*, asa*^+^*, ace*^+^*, gelE*^+^	*esp*^+^*, efbA*^+^*, asa*^+^*, ace*^+^*, cyl*^+^*, gelE*^+^	1
TET/MN	TET/MN	*esp*^+^*, efbA*^+^*, asa*^+^*, ace*^+^*, gelE*^+^	*esp*^+^*, efbA*^+^*, asa*^+^*, ace*^+^*, cyl*^+^*, gelE*^+^	1
TET/MN	TET/MN	*esp*^+^*, efbA*^+^*, asa*^+^*, ace*^+^*, cyl*^+^	*esp*^+^*, efbA*^+^*, ace*^+^	1
TET/MN	TET/MN	*esp*^+^*, efbA*^+^*, asa*^+^*, ace*^+^*, cyl*^+^*, gelE*^+^	*esp*^+^*, efbA*^+^*, ace*^+^*, cyl*^+^*, gelE*^+^	1
TET/MN	Sensitive to all antibiotics	*esp*^+^*, efbA*^+^*, asa*^+^*, ace*^+^*, cyl*^+^*, gelE*^+^	*esp*^+^*, efbA*^+^*, ace*^+^*, cyl*^+^*, gelE*^+^	1
TET/MN	TET/MN	*esp*^+^*, efbA*^+^*, ace*^+^*, cyl*^+^*, gelE*^+^	*esp*^+^*, efbA*^+^*, asa*^+^*, ace*^+^*, cyl*^+^*, gelE*^+^	1
TET/MN	TET/MN/GM(120)	*esp*^+^*, efbA*^+^*, asa*^+^*, ace*^+^*, cyl*^+^*, gelE*^+^	*esp*^+^*, efbA*^+^*, asa*^+^*, ace*^+^*, gelE*^+^	1
TET/MN	TET/MN	*esp*^+^*, efbA*^+^*, asa*^+^*, ace*^+^*, cyl*^+^*, gelE*^+^	*esp*^+^*, efbA*^+^*, ace*^+^*, gelE*^+^	1
TET/MN/GM(120)	TET/MN/GM(120)	*esp*^+^*, efbA*^+^*, asa*^+^*, ace*^+^*, cyl*^+^*, gelE*^+^	*esp*^+^*, efbA*^+^*, ace*^+^*, gelE*^+^	1
CP/GAT/LEV	TET/MN/CP/GAT/LEV	*efbA*^+^*, ace*^+^*, gelE*^+^	*esp*^+^*, efbA*^+^*, asa*^+^*, ace*^+^*, cyl*^+^*, gelE*^+^	1
TET/MN	Sensitive to all antibiotics	*esp*^+^*, efbA*^+^*, asa*^+^*, ace*^+^*, cyl*^+^*, gelE*^+^	*efbA*^+^*, ace*^+^*, gelE*^+^	1
TET/MN/GM(120)	TET/MN	*esp*^+^*, efbA*^+^*, asa*^+^*, ace*^+^*, cyl*^+^*, gelE*^+^	*efbA*^+^*, asa*^+^*, ace*^+^*, gelE*^+^	1
TET/MN	Sensitive to all antibiotics	*efbA*^+^*, ace*^+^*, gelE*^+^	*esp*^+^*, efbA*^+^*, asa*^+^*, ace*^+^*, cyl*^+^*, gelE*^+^	1
TET/MN	TET/MN	*esp*^+^*, efbA*^+^*, asa*^+^*, ace*^+^*, gelE*^+^	*esp*^+^*, efbA*^+^*, ace*^+^*, cyl*^+^*, gelE*^+^	1
TET/MN/GM(120)/CP/GAT/LEV	Sensitive to all antibiotics	*esp*^+^*, efbA*^+^*, asa*^+^*, ace*^+^*, cyl*^+^	*esp*^+^*, efbA*^+^*, ace*^+^*, gelE*^+^	1
GM(120)	Sensitive to all antibiotics	*esp*^+^*, efbA*^+^*, asa*^+^*, ace*^+^*, gelE*^+^	*ace*^+^	1
TET/MN	Sensitive to all antibiotics	*efbA*^+^*, ace*^+^*, gelE*^+^	*esp*^+^*, efbA*^+^*, asa*^+^*, cyl*^+^*, gelE*^+^	1
TET/MN/GM(120)/CP/GAT/LEV	Sensitive to all antibiotics	*efbA*^+^*, asa*^+^*, ace*^+^*, gelE*^+^	*ace*^+^*, gelE*^+^	1
Sensitive to all antibiotics	TET/MN	*efbA*^+^*, asa*^+^*, ace*^+^*, gelE*^+^	*esp*^+^*, efbA*^+^*, ace*^+^*, gelE*^+^	1
TET/MN/CP/GAT/LEV	TET/MN/CP/GAT/LEV	*efbA*^+^*, asa*^+^*, ace*^+^*, gelE*^+^	*efbA*^+^*, ace*^+^*, gelE*^+^	1
Sensitive to all antibiotics	Sensitive to all antibiotics	*efbA*^+^*, ace*^+^*, gelE*^+^	*efbA*^+^*, ace*^+^*, cyl*^+^*, gelE*^+^	1

+ , gene present

No. of patients: Number of patients; TET, tetracycline; MIN, minocycline; GM120, gentamicin 120 µg; CP, ciprofloxacin; LEV, levofloxacin; GAT, gatifloxacin; *esp,* Enterococcal surface protein; *asa1*, Aggregation substance; *ace*, Adhesion of collagen of enterococci; *cyl, *Cytolysin; *gelE*, Gelatinase; *efbA*, Pav A-like fibronectin-binding protein

## Discussion

Although the improvement of the sanitary and hygiene conditions limited the occurrence of some infections in the community, UTIs have remained common yet. *E. faecalis* isolates have been recognized as the second uropathogen in some countries [2, 3]. CA-UTI is a public health threat [6]; it can be mainly caused by *E. coli* and *Klebsiella* spp.; however, other bacteria, such as *Enterococcus, Proteus, Pseudomonas aeruginosa,* and *Staphylococcus* spp. can cause the infection similarly [10]. Most of these bacteria are members of the fecal microbiota and can cause the infection through an endogenous route [6, 11]. *E. faecalis* isolates have been recognized as the second uropathogen in some countries [2, 3].

In our study, we found a high prevalence of resistance to tetracycline and minocycline among *E. faecalis* strains in the urine and fecal specimens of symptomatic patients. This frequency was in agreement with the reports published by Maraki et al. and Ma et al. in Greece and China; [12, 13], but higher than the results obtained by other researchers from India, and Brazil [10, 14]. Arbitrary usage of antibiotics for the treatment of infections or agriculture could explain a higher rate of resistance to this antibiotic compared with other antimicrobials. In our study, the observed rates of resistance to tetracycline and minocycline in the fecal isolates were higher than those reported in the studies conducted by other researchers in healthy peoples [15–17]. This higher frequency of resistance among the fecal isolates could be caused by the possible transmission of Enterococci from animal reservoirs through the food chain. Link of Enterococci of animal origins with the strains colonizing the human intestine was described in several studies [18]. Since tetracycline might co-select vancomycin-resistant strains, special consideration should be done for enrichment or spread of these strains in humans. The frequency of resistance to gentamicin (120 µg), ciprofloxacin, levofloxacin, and gatifloxacin in urine specimens was 28.6%, 20.6%, 14.3% and 12.7%, which was relatively similar to those detected in fecal specimens (15.8%, 12.6%, and 6.3%, respectively). This rate is consistent with the report published by Sallem et al. in Tunisia, but lower than those reported by Tantry et al., Ma et al. and Linhares in India, China, and Portugal [2, 11, 12], and higher than the studies conducted by Novais et al. and Del Campo et al. in Portugal and Spain [15–17]. Penicillin G, ampicillin, vancomycin, nitrofurantoin, linezolid, and daptomycin were active against all the isolates from both types of the samples.

Due to the increase in MDR *E. faecalis* isolates, which has caused serious health concerns in HA-UTIs, there is little research on the phenotype of MDR these strains in CA-UTIs^23^. In our study, the low frequency of the MDR phenotype was detected in the strains isolated from urine and feces specimens (7.9% and 3.1%, respectively). This result was comparable to the 10% MDR rate reported in our previous study from CA-UTIs in Iran [19] but lower than those reported from patients with HA-UTIs [20] in urine specimens and CA-UTIs [21]. Also, this result was comparable to the 8.8% MDR rate reported by Hasannejad Bibalan and et al. in Iran [22] and lower than those reported from fecal healthy volunteers in Spain [15].

Several virulence determinants have been detected and examined among *E. faecalis* from different origins, such as clinical, food, and animal sources. However, there is little information about the relationship between their presence among different isolates and their capacity for tissue-specific pathogenicity [23]. Cross-contamination, through persistent colonization of the gastrointestinal tract as the main source of Enterococci, is considered as a source infection by *E. faecalis* in patients with CA-UTI [6]. Despite this possible involvement, there are no data about virulence entity of these strains to explain their capability for colonization and pathogenesis in the urinary tract, a phenomenon that was established for uropathogenic *E. coli* [6, 24].

In this study, *efbA, ace,* and *gelE* genes were the most prevalent virulence determinants in both types of samples. Our results were comparable to the results of Sharifi et al., Samadi Kafil et al., and Cosentino et al. among the isolates from patients with hospital-acquired UTIs High incidence of *efbA* in our isolates proposed this gene is important for virulence in UTIs [25–27]. EfbA, a PavA-like fibronectin-binding protein, plays an important role in adherence to extracellular matrix (ECM) proteins and is required for optimal virulence in an experimental model of ascending UTI [8]. Similarly, it seems that Ace protein (Adhesion to collagen of *E. faecalis*) bind to extracellular matrix proteins of the urinary tract, and plays an important role in early-stage colonization and pathogenesis of UTI [28], while gelatinase (*gelE*), is a secreted protease, that is involved in the dissemination of bacterium by the degradation of polymerized fibrin [29]. The frequency of *ace* and *gelE* genes in fecal specimens was higher than those reported from healthy volunteers in Tunisia [17]. In our study, the frequency of *esp, asa,* and *cyl* genes were 77.8%, 79.4%, and 54% in urine and 74.6%, 65%, and 46% in fecal specimens, respectively. Our result was similar previous reports published by other studies in HA-UTIs [26, 30–32] and in opposing with some other reports [25, 27]. In the case of *esp*, its frequency among our isolates was higher than those reported in Tunisia among *E. faecalis* isolates from healthy volunteers (25.4%) [17].

There is little information about multiple virulence determinants among *E. faecalis* isolates associated with CA-UTIs. *E. faecalis*, likely through multiple virulence factors that may involve in its colonization, survival, and pathogenicity, promote disease in the urinary tract. Heidari et al. investigated the incidence of genetic virulence markers among clinical *E. faecalis* and found that occurrence of multiple virulence factors was common in the urinary tract isolates, while most of the strains carried predominantly four, five and seven virulence determinants in HA-UTIs [33]. Aberna and Prabakaran investigated the presence of genetic virulence markers in *E. faecalis* and found that the occurrence of multiple virulence factors was common in the urinary tract isolates, while most of the strains carried predominantly two and three virulence determinants in HA-UTIs [24]. Shahraki and Rabi Nezhad Mousavi investigated the presence of seven virulence determinants in clinical multi-drug resistance Enterococci from patients with HA-UTI and found that most of the strains carried two virulence determinants in the urinary tract [34]. In the current study, 39.6% of the strains in urine specimens contained all the virulence determinants, while 28.5% and 25.3% carried five and four virulence determinants that were different from the aforementioned results. This discrepancy could be due to the difference in sample types and geographic locations; however, providing more accurate conclusions is not possible, since there is little information about community-acquired UTI through Enterococci and its association with related virulence determinants. Khalid investigated the occurrence of five virulence-associate genes in *E. faecalis* isolates associated with CA-UTIs and found that 28% of the strains contained all the virulence determinants, while 36% and 32% harbored four and five genetic markers of virulence [21]. In our previous study investigated the concomitant distribution of virulence genes among *E. faecalis* isolates and found that 28.5% of strains contain all virulence determinants, 28.5% and 30%, five, and four virulence determinants [19]. These results were in agreement with the current study results. In the current study, 36.5% of the strains in fecal specimens carried all the virulence determinants, while 19% and 31.7% contained five and four virulence determinants. Therefore the simultaneous presence of several virulence determinants in fecal specimens can enhance persistence and adhesion in the urinary tract. To have a better understanding about the link between carriage of the virulence determinants and resistance phenotypes, further study should be done at the expression level.

Results of RAPD-PCR showed that 26.9% of the isolates had similar molecular patterns in urine and fecal specimens in each patient, which was considered as endogenous strains. PFGE results also showed that 15.8% of the pair of isolates had similar pulsotypes in each patient. The importance of a complete virulence genotype in the occurrence of endogenous CA-UTIs was shown in our study. Accordingly, 64–70% of the isolates from patients with endogenous UTI showed complete genotype. These results showed that PFGE is a more reliable method and has better reproducibility than RAPD-PCR. Braak et al. examined two techniques of PFGE and RAPD-PCR on vancomycin-resistant *Enterococcus* (VRE) strains and similarly concluded that PFGE is a more reliable typing method [35]. The lower discriminatory power of RAPD-PCR compared with PFGE was reported by Barbier et al. for the study of VRE strains in hospitalized patients [36].

## Conclusions

In conclusion, our results provide the first report of molecular investigation for the detection of the endogenous source of CA-UTIs. Association between uropathogenic *E. faecalis* strains with the intestinal counterparts was shown based on their virulence and genomic background, which indicated that some intestinal strains could lead to CA-UTI due to the presence of some particular virulence determinants. Further studies are needed to determine genetic events that are involved in the acquisition or loss of these virulence genes. Susceptibility of these strains to most of the antimicrobials proposed administration of a different therapeutic strategy against the infection in these patients compared with those suffering from hospital-acquired UTIs in the clinical settings. Further studies on the urinary tract and gastrointestinal tract cell lines for investigation of the adherence capacity of the *E. faecalis* isolates will help us to better understand the contribution of these virulence factors in their colonization and pathogenesis.

## Methods

### Patients and bacterial strains

Through a clean catch method, 126 pairs of urine and stool specimens were collected from consecutive outpatients who attended to Milad hospital during August 2014 and March 2015 in Tehran, Iran. This study was approved by the ethics committee of Shahid Beheshti University of Medical Sciences. All patients provided written informed consent, similar to the Declaration of Helsinki before entry into the study. Samples of patients with a history of a recent hospitalization or antibiotic usage were excluded from the study. The freshly prepared urine specimens were inoculated on Sheep Blood agar using a calibrated loop and incubated at 37 °C for 24 h. Colony-forming units per milliliter ≥ 10^5^ was considered as bacteriuria. Fecal specimens were cultured on Enterococosel agar (BBL, USA) plates and incubated at 37 °C for 24 h. The presumed *E. faecalis* isolates in both samples were identified by the bacteriological conventional methods, including catalase, bile esculin test, fermentation of arabinose (1%), and growth in 6.5% NaCl solution [37]. PCR was performed with species-specific primers (see “Molecular examinations” section) to confirm the results of the biochemical tests. All the strains were stored at − 70 °C in Tryptic Soy Broth medium supplemented with 20% glycerol. *E. faecalis* ATCC 29212 was used as the control strain for both biochemical and molecular identification methods. To prevent the effect of mixed type infection, subcultures of a single colony from each sample was used for all the experiments.

## Molecular examinations

### Antimicrobial susceptibility testing

Antibiotic resistance phenotypes of the strains to 11 antibiotics were determined by disk diffusion (Kirby–Bauer) method according to the standard recommendation of the Clinical and Laboratory Standards Institute criteria [38]. Resistance to daptomycin was also tested using the E-test strip (Liofilchem^®^, Italy). *E. faecalis* ATCC 29212 and *Staphylococcus aureus* ATCC 25,923 were used as quality control strains. The antibiotic panels used were as follows: penicillin G (10 units), ampicillin (10 µg), vancomycin (30 µg), tetracycline (30 µg), minocycline (30 µg), ciprofloxacin (5 µg), levofloxacin (5 µg), gatifloxacin (5 µg), nitrofurantoin (300 µg), gentamicin (120 µg) and linezolid (30 µg) (Mast Group Ltd., United Kingdom).

### DNA extraction

High Pure PCR Template Preparation Kit (Roche, Germany) was used to extract genomic DNA from all Enterococci isolates with some modifications. Suspected colonies of Enterococci were subcultured onto the Blood agar medium. The grown colonies were mixed in 200 µl phosphate-buffered saline (PBS, pH, 8) and the pellets were resuspended in 200 µl PBS containing 5 µg lysozyme solutions and incubated at 37 °C for 15 min. The lysates were incubated with proteinase K (40 µl), and the obtained DNA samples were preserved at − 20 °C for polymerase chain reactions (PCR).

### Molecular characterization and virulence genotype determination of E. faecalis strains

Molecular characterization of *E. faecalis* was done by species-specific primers for *ddl*_*E.faecalis*_ gene (Table 7). The amplification was performed in a 25 μl reaction mixture containing 12.5 μl master Mix (Amplicon, Denmark), 10.5 μl distilled water, 0.5 μl of each of the primers (F and R), and 1 μl of template DNA. Specific primers were used to amplify sequences of *esp, efbA, asa, ace, cyl*, and *gelE* genes, as main virulence factors. PCR was performed in a thermal cycler (Eppendorf, Germany) at following conditions: initial denaturation at 95 °C for 5 min, followed by 35 cycles consisting of denaturation at 95 °C for 1 min, annealing ranging from 45 to 60 °C (Table 7) for 1 min, 72 °C for 1 min, and a final extension at 72 °C for 10 min. The PCR products were visualized using a UV transilluminator after electrophoresis in a 1% agarose gel and staining with the red safe solution (Bioneer, South Korea). To confirm the correct amplification of the target genes, direct sequencing of one amplified product for each gene was carried out using ABI 3730X capillary sequencer (Pishgam, Macrogen, Seoul, Korea).

**Table 7:** Oligonucleotide primers and conditions used to amplify different virulence marker genes in *E. faecalis* strains by PCR

Gene	Primer sequence (5′-3′)	Annealing temperature	Amplicon size (bp)	References
*ddl*_*E.faecalis*_	ATCAAGTACAGTTAGTCTTTATTAG ACGATTCAAAGCTAACTGAATCAGT	49	941	[19]
*Esp*	AGATTTCATCTTTGATTCTTGG AATTGATTCTTAGCATCTGG	48	510	[19]
*asa1*	TAGGAGTTGTAGGATTAGCTAC TGTTGTATTCMGCSACTTC	47	677	This study
*Ace*	GGAATGACCGAGAACGATGGC GCTTGATGTTGGCCTGCTTCCG	58	616	[12]
*cyl*	ACTCGGGGATTGATAGGC GCTGCTAAAGCTGCGCTT	52	688	[2]
*gelE*	TATGACAATGCTTTTTGGGAT AGATGCACCCGAAATAATATA	58	213	[2]
*efbA*	GCACAAGTCCCAAAAGGAGC AAGTGCGGCTTCAGTAAGGG	58	510	This study

*esp,* Enterococcal surface protein; *asa1*, Aggregation substance; *ace*, Adhesion of collagen of enterococci; *cyl, *Cytolysin; *gelE*, Gelatinase; *efbA*, Pav A-like fibronectin-binding protein

### Characterization of E. faecalis strains using DNA fingerprinting analysis techniques

#### Randomly amplified polymorphic DNA polymerase chain reaction (RAPD-PCR)

RAPD-PCR was performed using random primer 1283 (5′-GCGATCCCCA-3′) to screen genetic diversity among the *E. faecalis* strains. The amplification was performed in a 25 μl reaction mixture containing 12.5 μl master mix (Amplicon, Denmark), 8.5 μl of distilled water, 2 μM of primer, and 2 μl of template DNA. PCR was performed in a thermal cycler (Eppendorf, Germany) for cycles as follows: initial denaturation step at 94 °C for 4 min followed by 4 cycles consisting of denaturation (94 °C for 4 min), annealing (36 °C for 4 min), and extension (72 °C for 4 min), followed by a new cycle include denaturation (94 °C for 30 s), annealing (36 °C for 1 min), and extension (72 °C for 2 min) for 40 cycles and a final extension step at 72 °C for 10 min. Gel electrophoresis was used to interpret the results as described by [39]. The similarity of all banding profiles was analyzed by the GelCompar II software. *E. faecalis* ATCC 29212 was used as the control strain in this assay.

#### Pulsed-field gel electrophoresis (PFGE)

Genomic DNA was prepared in agarose plugs as described by Turabelidze et al. with some modifications [40]. In brief, after cell lysis by lysozyme and then incubation with proteinase K, DNA was digested with *Sma* I. The PFGE procedure was carried out using a contour-clamped homogeneous electric field apparatus (CHEF DRII, Bio-Rad Laboratories, USA). Digested genomic DNA of *Salmonella enterica serotype* Braenderup (H9812) was used as a size marker. The PFGE patterns were determined using the Dice coefficient in GelCompar II version 2.0 (Applied Maths, Belgium). Accordingly, isolates that differed by ≤ 3 bands were assigned to the same pulse-type (PT), while isolates that differed by ≥ 4 bands were assigned to different types [39].

### Statistical analysis

SPSS software version 17.0 (IBM SPSS Statistic) was used for statistical analysis.

## Data Availability

Data supporting the findings of this study of infectious diseases and the Center for Tropical Medical Research are available. However, there are restrictions on the availability of this data and are therefore not available to the public. However, the data are available at the request of the authors with a reasonable request and with permission from the infectious diseases and tropical medicine research center.

## Abbreviations

CA-UTI: Community acquired-urinary tract infection
HA-UTI: Hospital acquired-urinary tract infection
ECM: Extracellular matrix
*esp*: Enterococcal surface protein
*asa1*: Aggregation substance
*ace*: Adhesion of collagen of enterococci
*cyl*: Cytolysin
*gelE*: Gelatinase
*efbA*: Pav A-like fibronectin-binding protein
RAPD-PCR: Randomly amplified polymorphic DNA polymerase chain reaction
PFGE: Pulsed-field gel electrophoresis
